# Exogenous gallic acid enhances growth, essential oil accumulation, and antioxidant defense of dill under soil moisture deficit

**DOI:** 10.1186/s12870-026-10113-8

**Published:** 2026-11-12

**Authors:** Heba M. Gad, Aisha M. A. Ahmed, Hend E. Wahba, Khalid A. Khalid

**Affiliations:** 1 Medicinal and Aromatic Plants Department, National Research Centre, El Buhouth St, Dokki, 12622 Cairo Egypt; 2 Botany Department, National Research Centre, El Buhouth St, Dokki, 12622 Cairo Egypt

**Keywords:** Gallic acid, Field capacity, Yield, Essential oil, Monoterpenes

## Abstract

**Objective:**

Dill (*Anethum graveolens* L.) is a valuable aromatic plant, but its cultivation in Egypt’s sandy reclaimed soils is constrained by rapid soil moisture depletion. This study evaluated the effects of gallic acid (GA) on dill growth and biochemical responses under different moisture regimes.

**Methods:**

Plants were grown at four field capacity (FC) levels (100, 80, 60, and 40%) and either left untreated or foliar-sprayed with GA (30 mg L⁻¹). Morphological, biochemical and essential oil characteristics were evaluated, and data were analyzed using two-way analysis of variance (ANOVA).

**Key results:**

GA application improved biomass under full FC, increasing fresh and dry mass by 35.1% and 41.3%, respectively, compared with untreated plants at the same FC level. GA also enhanced essential oil production, with essential oil yield increasing by 70.3% at 60% FC compared with the corresponding untreated plants. At 40% FC, GA increased phenolic and flavonoid contents and DPPH radical-scavenging activity, with DPPH inhibition increasing by 10.7%. GA also promoted the accumulation of proline and antioxidant enzymes under severe moisture deficit.

**Conclusion:**

Foliar GA application enhanced biomass production, essential oil productivity, and antioxidant capacity in dill under reduced water availability, demonstrating its potential as a biostimulant for improving dill performance under moisture-deficit conditions.

**Graphical Abstract:**

**Figure:**
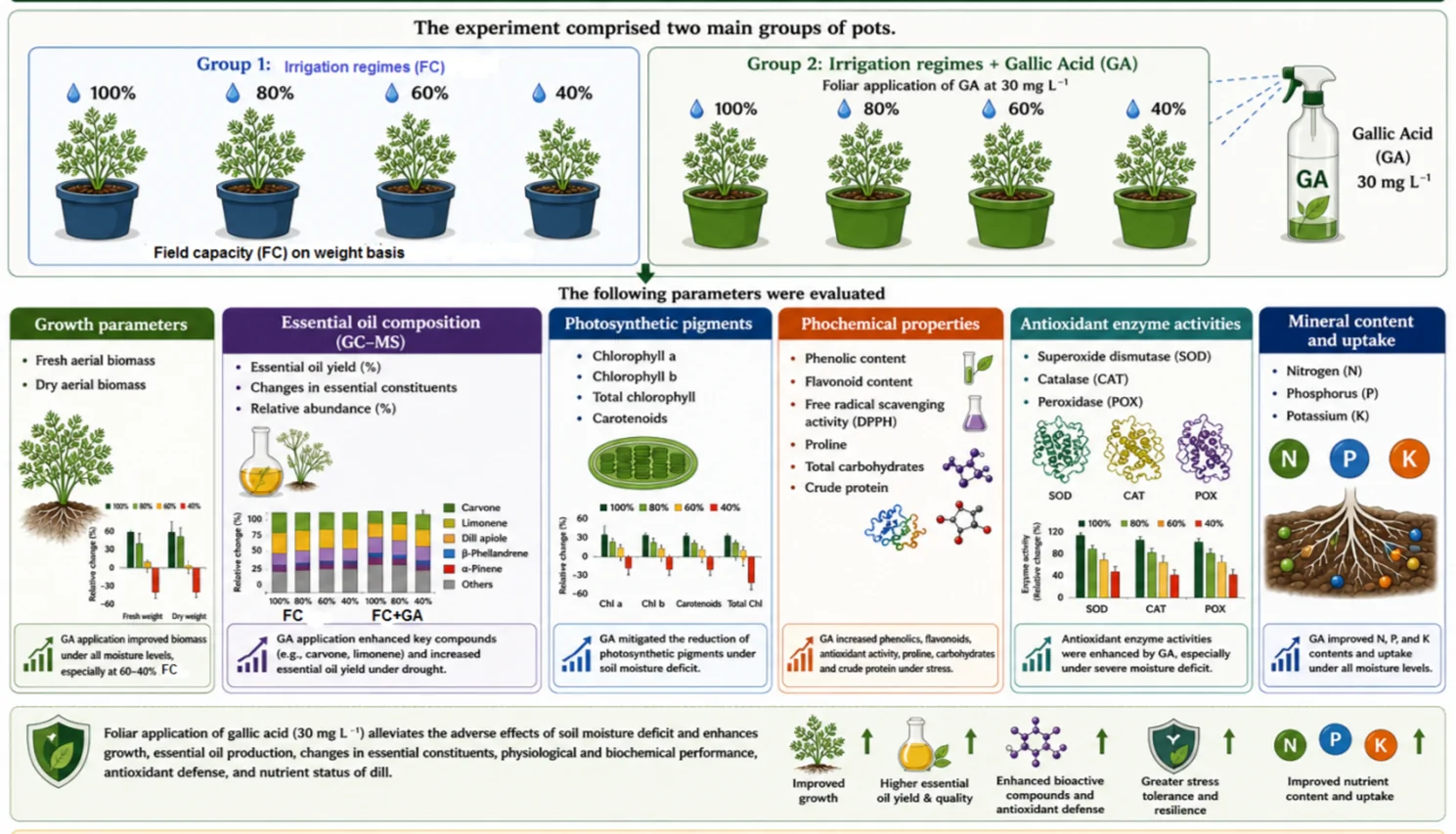
Effect of gallic acid (GA) and field capacity (FC) on the dill productivity

## Introduction

Aromatic plants are valuable sources of bioactive compounds, including essential oils, phenolic compounds, flavonoids, and other secondary metabolites, which possess diverse biological activities and industrial applications [1]. Due to their antioxidant, antimicrobial, anti-inflammatory, and preservative properties, these natural products have gained considerable importance in the pharmaceutical, nutraceutical and food industries as sustainable alternatives to synthetic additives. Among aromatic plants, dill (*Anethum graveolens* L.), a member of the Apiaceae family, is widely cultivated for its culinary, medicinal, and industrial value. Its leaves are consumed fresh or dried as a flavoring herb, while its fruits and essential oil are important sources of aromatic and bioactive constituents used in food, pharmaceutical, and related industries. Dill essential oil and phenolic compounds contribute to its characteristic aroma and have been associated with several biological activities, including antioxidant, antimicrobial, antispasmodic, and anti-inflammatory effects. These diverse applications highlight the multifunctional and commercial importance of dill as a crop [1, 2]. Consequently, the productivity and quality of dill depend not only on biomass production but also on the accumulation and total yield of essential oil and other bioactive compounds. Production conditions, particularly water availability, can substantially affect both vegetative growth and secondary metabolite accumulation. Therefore, simultaneous evaluation of plant biomass, essential oil content, and essential oil yield is important for assessing the overall productivity and practical value of dill under different growing conditions. Notably, an increase in essential oil concentration does not necessarily result in a higher essential oil yield, as total oil production is also determined by the amount of biomass produced [1, 2].

Insufficient moisture availability is a key environmental factor that negatively impacts plant growth, yield, and quality [3–5]. Moisture deficit stress (MDS) can adversely affect plants at any stage of development by disrupting numerous physiological and biochemical processes. These effects include reductions in chlorophyll content, photosynthetic carbon assimilation, nutrient uptake, and hormonal balance, as well as alterations in source sinks relationships. Furthermore, MDS impairs xylem and phloem transport, compromises cell membrane stability, and causes osmotic disturbances, ultimately leading to reduced plant growth and overall performance [6–8]. Moreover, MDS markedly influences the production of reactive oxygen species (ROS), leading to the accumulation of both free radical species, such as hydroxyl, alkoxy, and superoxide radicals, and non-radical molecules, including hydrogen peroxide and singlet oxygen [4, 9, 10]. Excessive accumulation of reactive oxygen species (ROS), whether directly or indirectly induced, can cause substantial damage to DNA, RNA, and proteins. As a result, cellular metabolism and organelle functions are impaired, leading to significant reductions in plant growth and productivity [11, 12]. Sustained MDS adversely affects the development of aerial plant organs, decreases leaf area available for transpiration, and promotes early senescence. Under severe conditions, continued MDS may culminate in irreversible damage and plant mortality [13, 14]. Generally, MDS triggers antioxidant defense systems comprising enzymatic and non-enzymatic components that protect plant cells from ROS-mediated oxidative injury [10, 15]. The antioxidant defense system that contributes to drought tolerance comprises enzymatic antioxidants, such as catalase (CAT), peroxidase (POD), superoxide dismutase (SOD), and ascorbate peroxidase (APX), as well as non-enzymatic antioxidants, including ascorbate, reduced glutathione (GSH), carotenoids, and various tocopherol compounds [16, 17]. Besides antioxidant defense mechanisms, various osmolytes play a crucial role in enhancing MDS tolerance. These include proline, quaternary ammonium compounds such as glycine betaine, amino acids, sugars, proteins, and other carbohydrates, which help maintain cellular osmotic balance under MDS [18]. Furthermore, the exogenous application of osmoprotectants and antioxidants has been shown to effectively mitigate the adverse effects of MDS. For instance, the external application of ascorbic acid has demonstrated considerable potential in enhancing plant tolerance to drought conditions [3], alpha-tocopherol [19], trehalose for plant [20, 21] and thiamin [4] under MDS, resulting in enhanced growth and greater yield production.

Biostimulants are bioactive compounds that represent an innovative biotechnological approach for modulating plant metabolism and enhancing crop performance. Their application stimulates key physiological processes, including photosynthetic efficiency, nutrient assimilation, hormonal regulation, and antioxidant defense systems, thereby improving plant growth, productivity, and stress tolerance. In aromatic and medicinal plants, biostimulants can significantly influence the biosynthesis, yield, and chemical composition of essential oils and other valuable secondary metabolites, even when applied at low concentrations. These effects contribute to the accumulation of high-value natural products with important applications in the pharmaceutical, food, and cosmetic industries [22, 23]. Previous research has demonstrated that exogenous application of biostimulants can enhance the biosynthesis, yield, and composition of essential oils and their constituents [24]. In response to the growing demand for sustainable and environmentally friendly agricultural practices, biostimulants have emerged as a promising tool for improving essential oil production through safe and eco-compatible approaches. Consequently, the biostimulant industry is expected to experience continued growth in the coming years due to its role in promoting plant physiological performance and the accumulation of valuable secondary metabolites [25].

Phenolic compounds are among the most important bioactive substances involved in plant adaptation to biotic and abiotic stresses. Acting as natural biostimulants, they can improve plant physiological performance, nutrient uptake and utilization, growth, and stress tolerance [25]. Among these compounds, Gallic acid (GA) has attracted considerable attention because of its potent antioxidant activity and potential role in stress mitigation. GA can enhance plant resilience by scavenging reactive oxygen species (ROS), stimulating antioxidant defense systems, reducing lipid peroxidation, maintaining membrane stability, improving osmotic adjustment, and preserving photosynthetic efficiency, particularly under drought stress [26, 27]. Exogenous GA application has also been reported to improve plant tolerance to cold stress by enhancing antioxidant activity, maintaining plant water status, and promoting photosynthetic efficiency [28]. In plant callus cultures, GA mitigated boron toxicity by reducing boron accumulation and alleviating growth inhibition, thereby supporting normal plant development [29]. Furthermore, GA treatment has been shown to improve salinity tolerance by stimulating growth, increasing chlorophyll content, enhancing the accumulation of glutathione and phenolic compounds, and regulating antioxidant defense and ion homeostasis [26]. In aromatic plants, exogenous phenolic compounds may also influence essential oil yield and chemical composition, potentially enhancing their biological properties and product quality [24]. Therefore, GA represents a promising biostimulant for improving plant performance, stress tolerance, and the production of valuable secondary metabolites under adverse environmental conditions, although further field studies are needed to validate its effectiveness under practical growing conditions [26, 27].

Due to the limited availability of agricultural land in Egypt, which accounts for only about 4% of the country’s total area and is insufficient to meet the demands of its growing population, considerable efforts have been directed toward the reclamation and cultivation of sandy soils, which cover large areas of the country [30]. These soils are predominantly distributed in arid and semi-arid regions and generally have low water-holding capacity, rapid infiltration and drainage, and limited retention of organic matter and nutrients. Consequently, water and nutrient availability in the root zone can decline rapidly, particularly under water-limited conditions, posing important challenges for the cultivation of aromatic crops. Modern irrigation systems, including drip and sprinkler irrigation, are therefore widely used to improve water-use efficiency and reduce water losses. However, complementary approaches such as the application of GA may further support plant performance under limited water availability by potentially enhancing physiological and biochemical responses associated with drought tolerance [31]. For dill production, these soil characteristics may affect plant water status, nutrient uptake, growth, and biomass accumulation and may consequently modify physiological and biochemical processes associated with secondary metabolite production. Since water and nutrient availability can influence essential oil biosynthesis and accumulation, cultivation of dill in sandy soils may affect both essential oil concentration and total yield. Understanding these responses is therefore important for optimizing dill biomass and essential oil productivity under the water-limited conditions prevailing in reclaimed sandy soils [30, 31].

Despite the reported beneficial effects of GA in alleviating various abiotic stresses, its potential to improve dill performance under water limitation, particularly in sandy soils, remains insufficiently understood. Therefore, the present study investigated the interactive effects of water availability, expressed as field capacity (FC), and foliar GA application on dill growth, physiological and biochemical responses, antioxidant activity, nutrient status, and essential oil production. Particular attention was given to growth traits, photosynthetic pigments, phenolic compounds, flavonoids, antioxidant activity, nutrient accumulation, and essential oil content, yield, and composition. The study also examined whether GA could modify plant responses to water limitation and whether changes in essential oil concentration were accompanied by corresponding changes in total essential oil yield. We hypothesized that exogenous GA would enhance plant performance under water limitation by improving physiological and biochemical responses and maintaining essential oil productivity. As the experiment was conducted under controlled greenhouse conditions, the findings provide a proof-of-concept that requires further validation under open-field conditions before broader agronomic recommendations can be made.

## Materials and methods

### Experimental procedure

The experiment was conducted in a greenhouse at the National Research Centre, Egypt, over two consecutive growing seasons (2023/2024 and 2024/2025). Throughout the experimental period, the greenhouse was maintained at 29/14°C (day/night) and 70/50% relative humidity (day/night). Plants were exposed to natural daylight and the naturally changing photoperiod throughout each growing season, without supplemental artificial lighting. The photosynthetic photon flux density (PPFD) inside the greenhouse was approximately 3700 µmol m⁻² s⁻¹ during daytime, as measured using a VidalKraft Quantum PAR Meter (Model VK131). Dill seeds were obtained from the Institute of Medicinal and Aromatic Plants, Agricultural Research Centre, Egypt. Uniform seeds were sown in plastic pots measuring 30 cm in diameter and 50 cm in height. During the first week of November in both seasons, the pots were transferred to a greenhouse under natural environmental conditions. Each pot was filled with 10 kg of air-dried sandy soil. Three weeks after sowing, seedlings were thinned to retain three plants per pot. Experiment comprised two main groups of pots. First group was exposed to different irrigation regimes corresponding to 100, 80, 60, and 40% of field capacity (FC) on a weight basis. Second group received the same irrigation treatments, along with foliar application of GA at 30 mg L^− 1^. GA concentration was selected exclusively based on preliminary trials, which indicated its potential effectiveness in improving plant responses under the experimental conditions. All agronomic practices were carried out in accordance with standard recommendations of Egyptian Ministry of Agriculture. All agronomic practices were carried out in accordance with the standard recommendations of the Egyptian Ministry of Agriculture. Compost was incorporated into the soil at a rate of 10 g pot⁻¹ prior to planting. Chemical fertilizers were applied at rates of 6 g ammonium sulfate (21.5% N), 4 g calcium superphosphate (15.5% P₂O₅), and 2 g potassium sulfate (50% K₂O) pot⁻¹, supplying approximately 1.3 g N, 0.6 g P₂O₅, and 1 g K₂O pot⁻¹, respectively. Phosphorus fertilizer was applied as a basal dose before sowing, while nitrogen and potassium fertilizers were applied in split doses according to the recommended schedule for the crop growth stages. The same soil was used for all treatments, ensuring uniform initial nutrient and organic matter conditions across the experimental units. Therefore, differences in plant nutrient status were evaluated under the imposed FC × GA treatments without additional fertilizer inputs. Physical and chemical properties of the soil used in this study were determined following the methods of Jackson [32] and Cottenie [33], and results are presented in Table 1. Field capacity (FC) was determined gravimetrically by saturating the soil with water, allowing excess water to drain, and recording the pot weight at field capacity. Throughout the experiment, pots were weighed regularly, and the amount of water lost was replenished to maintain the assigned FC level for each treatment. GA solutions were prepared in 1000 mL volumetric flasks by dissolving 0.03 g of pure GA in a small volume of ethanol. The solution was then brought up to a final volume of 1000 mL using distilled water. GA was applied as a foliar spray in two applications: the first was conducted two weeks after thinning, while the second application was carried out two weeks following the first.

**Table 1:** Characterization of the experimental soil

Constituents and Characteristics	Rates
Coarse sand (%)	14.4
Fine sand (%)	80.1
Silt fraction **(%)**	3.1
Clay fraction (%)	2.4
pH (soil: solution, 1:2.5)	7.9
Electrical conductivity (EC) (dS m^− 1^)	1.8
Organic matter (OM) (%)	0.2
Nitrogen content (%)	0.2
Total phosphorus (mg 100 g^− 1^ Soil)	2.3
Cation composition (mg 100 g^− 1^)	
Potassium, K	4.2
Iron (Fe)	1.5
Manganese, Mn	3.3
Zinc, Zn	3.8
Copper, Cu	2.6
Calcium, Ca	28.4
Magnesium, Mg	7.2
Sodium, Na	33.9
Anion concentrations (mg 100 g^− 1^)	
Calcium carbonate, CaCO_**3**_	16.7
Chloride, Cl	27.5
Carbonate (CO₃²⁻)	24.2
Sulfate, SO_4_	13.6
Nitrates, NO_3_	2.9

### Harvesting

Dill plants were collected at full bloom during both seasons, 90 days after sowing, by cutting the plants approximately 5 cm above the soil surface. Fresh and dry herb weights were measured and recorded on a per-plant basis (g plant^− 1^).

### Photosynthetic pigment determination

The contents of chlorophyll *a*, chlorophyll *b*, and carotenoids were assessed in fresh leaf samples from each treatment following the procedure outlined by Arnon [34]. Fresh leaf tissues were thoroughly homogenized in 80% acetone using a mortar and pestle. The absorbance of the extracts was then measured using a spectrophotometer (Shimadzu UV-1700, Tokyo, Japan) at wavelengths of 663 nm and 645 nm for chlorophyll *a* and chlorophyll *b*, respectively, and 470 nm for carotenoids. The concentrations of photosynthetic pigments were calculated and expressed as mg 100 g^− 1^ fresh weight (FW).Chlorophyll *a* and chlorophyll *b* were calculated as follows: Chl *a* = 12.25 A₆₆₃ − 2.79 A₆₄₅ and Chl *b* = 21.50 A₆₄₅ − 5.10 A₆₆₃. Total carotenoids were calculated using the equation: Carotenoids = [1000 A₄₇₀ − 2.27(Chl *a*) − 81.4(Chl *b*)]/227.

### Essential oil extraction

Fresh leaf samples from each treatment were prepared in three replicates. Essential oil was extracted by hydro distillation using a Clevenger-type apparatus following the method of Clevenger [35]. Briefly, 100 g of dried leaf material from each replicate was subjected to hydro distillation for 3 h, duration determined in preliminary trials to ensure complete oil recovery. The plant material was placed in a 2 L round-bottom flask containing 1 L of distilled water and heated to boiling (100 °C). The extracted essential oil was collected and dried over anhydrous sodium sulfate to remove residual moisture. The oil samples were then stored in sealed vials at 4 °C until further analysis. Corresponding dry weight of each fresh sample was determined separately for each treatment and used to express essential oil content (%, w/w), while essential oil yield was calculated on the basis of the plant dry weight (mg plant^-1^). The essential oil was extracted from fresh leaf material, while parallel subsamples from the same treatment were dried separately to determine the corresponding dry weight used for expressing essential oil content and yield on a dry-weight basis.

### Essential oil analysis

The chemical composition of the essential oil was analyzed using a Varian 3400 GC–MS system equipped with a DB-5 fused silica capillary column (60 m × 0.25 mm i.d., 0.25 μm film thicknesses). The oven temperature was programmed from 50 °C to 240 °C at a rate of 4 °C min⁻¹. Helium was used as the carrier gas at a linear velocity of 31.5 cm s⁻¹. The transfer line temperature was maintained at 260 °C. Samples were injected with a split ratio of 1:60. Mass spectra were obtained under electron ionization (EI) mode at 70 eV. The mass spectrometer was operated with a scan time of 1 s and a mass scan range of 40–300 m/z. Identification of the constituents was achieved by comparing their mass spectra and retention indices with those reported in the NIST and Wiley spectral libraries and published literature. Compound identities were further confirmed through comparison of their retention indices (RI) with published literature values or with those of authentic reference standards [36].

### Evaluation of phenols

Leaves were washed with distilled water, shade-dried to constant weight, and finely ground. A 1-g portion of the powder was extracted with 10 mL of 80% methanol (1:10, w/v) by shaking for 24 h at room temperature. The extract was filtered through Whatman No. 1 filter paper and kept at 4 °C until analysis. According to Singleton [37], total phenolic content in leaf samples was determined using the Folin–Ciocalteu method. Briefly, 2 mL of crude extract (1 mg mL^− 1^) was diluted with distilled water to a final volume of 3 mL, then mixed thoroughly with 0.5 mL of Folin–Ciocalteu reagent and allowed to react for 3 min. After that, 2 mL of 20% (w/v) sodium carbonate solution was added to the mixture. The reaction mixture was incubated in the dark for 60 min, after which absorbance was recorded at 650 nm. Total phenolic content was calculated using a gallic acid calibration curve and expressed as milligrams of gallic acid equivalents per gram of dry weight (mg GAE/g DW).

### Estimation of flavonoids

Dried leaves were pulverized, and 1 g of the resulting powder was extracted with 10 mL of 80% methanol (1:10, w/v) by shaking at room temperature for 24 h. The extract was subsequently filtered through Whatman No. 1 filter paper and kept at 4 °C until further analysis. According to Pourmorad [38], total flavonoid content in leaf samples was determined using the aluminum chloride colorimetric method. Briefly, 50 µL of crude extract (1 mg mL^− 1^ in ethanol) was adjusted to 1 mL with methanol, and then mixed with 4 mL of distilled water. Subsequently, 0.3 mL of 5% sodium nitrite (NaNO₂) solution was added and the mixture was incubated for 5 min. After that, 0.3 mL of 10% aluminum chloride (AlCl_3_) solution was introduced and allowed to stand for 6 min. Next, 2 mL of 1 mol L^− 1^ sodium hydroxide (NaOH) was added, and the final volume was adjusted to 10 mL using double-distilled water. The reaction mixture was left for 15 min, and absorbance was then recorded at 510 nm. Total flavonoid content was calculated using a rutin calibration curve and expressed as milligrams of rutin equivalents per gram of dry weight (mg RE g DW^− 1^).

### Evaluation of antioxidant activity

Dried leaf samples were finely powdered, and 1 g was extracted with 10 mL of 80% methanol (1:10, w/v) under shaking for 24 h at room temperature. The extract was filtered through Whatman No. 1 filter paper and stored at 4 °C until DPPH analysis. Antioxidant activity of leaf extracts at the full flowering stage was evaluated using the 1,1-diphenyl-2-picrylhydrazyl (DPPH) radical scavenging assay, as described by Middha [39]. In brief, 200 µL of each extract at different concentrations (100–500 µg mL^-1^) were mixed with 3.8 mL of DPPH solution and incubated in the dark at room temperature for 1 h. Absorbance of the reaction mixture was then measured at 517 nm. Ascorbic acid was used as a positive control. The ability of the samples to scavenge DPPH free radicals was determined accordingly. The DPPH radical scavenging activity was calculated using the following equation:

DPPH inhibition (%) = [(A₀ − A₁) / A₀] × 100; where A₀ is the absorbance of the control and A₁ is the absorbance of the sample.

### Crude protein analysis

According to Jones [40], crude protein content in leaf samples was determined using the micro-Kjeldahl method. Approximately 1 g of dried plant material was digested with 15 mL of concentrated sulfuric acid (H_2_SO_4_) in the presence of two copper catalyst tablets using a Kjeltec System 2020 digestor (Tecator Inc., Herndon, VA, USA) at 420 °C for 2 h. After digestion and cooling, distilled water was added to the hydrolysate prior to neutralization and subsequent titration. Total nitrogen content was then converted to crude protein by multiplying by the standard factor of 6.25.

### Determination of carbohydrates

Total carbohydrate content in dried leaf samples from each treatment was estimated using the method of Dubois [41]. For extraction, foliar tissues were homogenized in 1 N H_2_SO_4_. The resulting extract was then analyzed spectrophotometrically by recording absorbance at 490 nm. D-glucose was employed as the standard reference for calibration and quantification.

### Proline determination

The proline content was quantified following the procedure of Bates [42]. Fresh leaf tissue (0.5 g) was homogenized in 10 mL of 3% sulfosalicylic acid and subsequently filtered. An aliquot of 2 mL of the filtrate was reacted with 2 mL of acid ninhydrin and 2 mL of glacial acetic acid. The reaction mixture was heated in a boiling water bath (100 °C) for 1 h and then immediately cooled in an ice bath to stop the reaction. Thereafter, 4 mL of toluene were added to extract the reaction product, and the upper toluene layer was carefully separated. Absorbance was recorded at 520 nm using a spectrophotometer. Proline concentration was determined using a standard calibration curve and expressed as µmol g^− 1^ FW.

### Investigation of antioxidant enzyme activity

***Extraction***: The method adopted in enzyme extraction was that described by Mukherjee [43]. A fresh plant tissue (2 g) were frozen in liquid nitrogen and finely grounded by pestle in a chilled mortar. The enzymes were extracted in 100 mM phosphate buffer (pH 6.8) containing KH_2_PO_4_ and K_2_HPO_4_. The homogenates were centrifuged at 20,000_×g_ for 20 min. The supernatant was made up to a known volume with the same buffer and used as enzyme preparation for assaying the activity of certain enzymes. ***Assay of catalase activity (CAT) EC 1.11.1.6***: CAT activity was assayed according to the method of Kar [44]. The enzyme extract (40 µl) was added to 9.96 ml of H_2_O_2_ in phosphate buffer at pH 7.0 (0.16 ml of 30% H_2_O_2_ in 100 ml of 50 mM phosphate buffer). CAT activity was determined by measuring the rate of change of H_2_O_2_ absorbance within 1 min with UV-spectrophotometer (Spectronic 601) at 250 nm. One unite of enzyme activity was defined as the amount of the enzyme that reduced 50% of H_2_O_2_ in 1 min at 25 °C. The enzyme activity was expressed unit g^-1^ FW min^-1^. ***Assay of superoxide dismutase activity (SOD) EC 1.15.1.1***: SOD activity was determined by measuring the inhibition of the auto-oxidation of pyrogallol using a method described by Marklund [45]. 10 ml of the reaction mixture comprised: 3.6 ml of distilled water, 0.1 ml of the enzyme extract, 5.5 ml of 50 mM phosphate buffer (pH 7.8) and 0.8 ml of 3 mM pyrogallol (dissolved in 10 mM HCl). The rate of pyrogallol reduction was measured at 325 nm using UV-spectrophotometer (Spectronic 601). One unit of enzyme activity was defined as the amount of the enzyme that resulted in 50% inhibition of the auto-oxidation rate of pyrogallol at 25 ˚C. The enzyme activity was expressed unit g^-1^ FW min^-1^. ***Assay of peroxidase activity (POX) EC 1.11.1.7***: POX activity was assayed following the method of Kar [44] with slight modifications. Five ml of the assay mixture contained 300 µM of phosphate buffer (pH 6.8), 50 µM catechol, 50 µM H_2_O_2_ and 1 ml of crude enzyme extract were prepared. After incubation at 25 ˚C for 5 min, the reaction was stopped by the addition of 1 ml of 10% H_2_SO_4_. The optical density was recorded at 340 nm by using spectrophotometer (Spectronic 601, Milton Roy Company). The enzyme activity was expressed unit g^-1^ FW min^-1^.

### Elemental analysis

For elemental analysis, plant leaves were washed thoroughly with distilled water, dried at 70 °C to constant weight, and finely ground. A 0.5 g portion of the powdered sample was subjected to wet digestion using a mixture of concentrated HNO₃ and HClO₄. The digested samples were cooled, filtered, and diluted to a known volume with distilled water. Nitrogen (N) content was analyzed using the micro-Kjeldahl method as described by Jones [40]. Phosphorus (P) concentration was measured spectrophotometrically according to the procedure of Snell [46]. Potassium (K) content was estimated using a flame photometer following the method outlined by Chapman [47].

### Evaluation and analysis of data

The experiment was conducted as a two-factor factorial arrangement within a randomized complete block design (RCBD), with four FC levels (100, 80, 60, and 40% FC) and two GA concentrations (0 and 30 mg L⁻¹). Each block contained all eight FC × GA treatment combinations, with three blocks serving as independent experimental replicates. The 10 pots within each replicate were considered subsamples; therefore, their mean value was calculated and used as a single experimental observation for statistical analysis [48]. The statistical model used was Yijk = µ + Bi + Fj + Gk + (FG)jk + εijk, where Yijk represents the observed value of the response variable, µ is the overall mean, Bi represents the block effect, Fj represents the effect of the FC level, Gk represents the effect of the GA concentration, (FG)jk represents the interaction effect between FC and GA, and εijk represents the random experimental error.

Data from each growing season were initially analyzed to assess the effects of FC, GA, and their interaction, as well as the effect of growing season and its interactions with the experimental factors. The season × FC, season × GA, and season × FC × GA interactions, as well as the main effect of season, were not statistically significant. Therefore, data from the two growing seasons were pooled for subsequent analysis.

Prior to analysis of variance (ANOVA), the normality of model residuals and homogeneity of variance were assessed using the Shapiro - Wilk and Levene’s tests, respectively. Both assumptions were satisfied, and no data transformation was required. The pooled data were subjected to two-factor factorial ANOVA within an RCBD, with block included in the model as a source of variation. The statistical model included the effects of block, FC, GA, and the FC × GA interaction. Based on 24 experimental observations, the degrees of freedom were 2 for block, 3 for FC, 1 for GA, 3 for the FC × GA interaction, and 14 for experimental error, with 23 total degrees of freedom. Statistical significance was assessed at *P* ≤ 0.05, while *P* ≤ 0.001 was considered highly significant. When significant differences were detected, Duncan’s multiple range tests was used for mean separation at *P* < 0.05. Statistical analyses were performed using STAT-ITCF (version 7) according to Foucart [49].

## Results

### Response of dill growth parameters to FC, GA, and their interactions

FC and GA affected plant morphology (Table 2). Plant growth parameters, including total fresh mass, and total dry mass, were influenced by FC levels and GA application. Overall, decreasing FC progressively reduced all measured growth parameters, with the greatest reductions recorded at 40% FC. In contrast, GA application alleviated the adverse effects of FC treatments and improved plant growth across FC levels. The highest values of total fresh mass (35 g plant^− 1^) and total dry mass (11.3 g plant^− 1^) were obtained under full FC combined with GA application. The differences on both morphological characters were non-significant for FC x GA, and they were highly significant (*P* ≤ 0.001) for FC or GA.

**Table 2:** Effect of gallic acid and / or field capacity on growth, photosynthetic pigments and essential oil production

Field capacity (%)	Gallic acid (mg L^− 1^)	Mass production (g plant^− 1^)		Photosynthetic pigments (mg 100 g^− 1^)			Essential oil production	
		Fresh	Dry	Chlorophyll		Carotenoids	(%)	(mg plant^− 1^)
				*a*	*b*			
100	Without gallic acid	25.9 ± 1.5	8.0 ± 0.7	26.2 ± 0.9^c^	15.2 ± 0.8	18.7 ± 0.6	0.7 ± 0.1	55.7 ± 6.2
80		22.3 ± 2.2	6.6 ± 0.9	22.6 ± 0.7^de^	13.7 ± 1.0	16.4 ± 0.6	1.3 ± 0.3	87.7 ± 5.1
60		20.2 ± 2.2	6.1 ± 1.5	20.5 ± 1.0^ef^	11.6 ± 1.0	13.3 ± 0.8	1.7 ± 0.2	101.7 ± 6.8
40		17.1 ± 1.3	4.8 ± 0.5	18.6 ± 0.5^f^	9.7 ± 0.6	11.4 ± 0.6	2.1 ± 0.3	100.6 ± 7.6
Overall without gallic acid		21.4 ± 3.7^b^	6.4 ± 1.5^b^	22.0 ± 3.0^b^	12.6 ± 2.3^b^	15.0 ± 3.0^b^	1.5 ± 0.6^b^	86.7 ± 13.6^b^
100	With gallic acid	35.0 ± 1.5	11.3 ± 1.4	32.1 ± 0.7^a^	17.3 ± 0.9	21.9 ± 0.4	1.0 ± 0.3	115.4 ± 4.2
80		30.7 ± 1.3	9.4 ± 0.9	28.3 ± 1.0^b^	14.8 ± 0.5	19.8 ± 0.7	1.5 ± 0.3	139.2 ± 7.6
60		28.1 ± 2.4	8.8 ± 1.3	23.6 ± 0.8^d^	12.3 ± 0.7	15.5 ± 0.7	2.0 ± 0.4	173.2 ± 11.6
40		24.0 ± 1.6	5.9 ± 0.8	20.1 ± 0.5^ef^	10.8 ± 1.4	12.9 ± 0.4	2.4 ± 0.1	141.1 ± 13.3
Overall with gallic acid		29.5 ± 4.4^a^	8.9 ± 0.8^a^	26.0 ± 4.8^a^	13.8 ± 2.6^a^	17.5 ± 3.7^a^	1.7 ± 0.6^a^	142.2 ± 29.7^a^
100	Overall field capacity	30.5 ± 5.2^a^	9.7 ± 2.1^a^	29.2 ± 3.3^a^	16.3 ± 1.4^a^	20.3 ± 1.8^a^	0.9 ± 0.2^d^	86.3 ± 14.6^b^
80		26.5 ± 4.9^b^	8.0 ± 1.8^b^	25.5 ± 3.2^b^	14.3 ± 0.9^b^	18.1 ± 1.9^b^	1.4 ± 0.3^c^	113.5 ± 8.9^ab^
60		24.2 ± 8.7^c^	7.5 ± 1.9^b^	22.1 ± 1.9^c^	12.0 ± 0.8^c^	14.4 ± 1.4^c^	1.9 ± 0.3^b^	137.5 ± 10.7^a^
40		20.6 ± 4.0^d^	5.4 ± 0.8^c^	19.4 ± 0.9^d^	10.3 ± 0.8^d^	12.2 ± 1.0^d^	2.3 ± 0.2^a^	121.3 ± 27.3^ab^
F values								
Gallic acid		120.3^**^	33.0^**^	163.3^**^	15.9^**^	108.3^**^	7.1^*^	36.4^**^
Field capacity		31.9^**^	16.9^**^	179.4^**^	69.9^**^	218.0^**^	34.1^**^	5.6^*^
Gallic acid x Field capacity		0.4^ns^	1.2^ns^	11.2^**^	0.9^ns^	3.2^ns^	0.1^ns^	0.5^ns^
P values								
Gallic acid		0.000	0.000	0.000	0.001	0.000	0.017	0.000
Field capacity		0.000	0.000	0.000	0.000	0.000	0.000	0.008
Gallic acid x Field capacity		0.759	0.334	0.000	0.461	0.051	0.981	0.663
MSE		3.3	1.1	0.6	0.6	0.4	0.1	508.9

F-statistics: * and ** indicate significance at *P* ≤ 0.05 and *P* ≤ 0.001, respectively; all values are presented as mean ± standard deviation (SD). MSE, mean squared error

### Effects of FC, GA, and their interaction on photosynthetic pigments of dill

Application of GA enhanced the accumulation of photosynthetic pigments such as Ch *a*, Ch *b* and total carotenoids (Table 2), However, the contents of these photosynthetic pigments decreased progressively with decreasing FC. The greatest contents of Ch *a* (32.1 mg 100 g^− 1^), Ch *b* (17.3 mg 100 g^− 1^), and total carotenoids (21.9 mg 100 g^− 1^) were recorded in plants treated with GA x full FC conditions. Ch *a* was highly significantly affected (*P* ≤ 0.001) by GA, FC, and their interaction. In contrast, Ch *b* and total carotenoids were highly significantly influenced (*P* ≤ 0.001) by the main effects of GA and FC, while the GA × FC interaction had no significant effect.

### Effects of FC, GA, and their interaction on essential Oil production of dill

As shown in Table 2, FC, GA, and their interaction affected the essential oil percentage and yield of dill plants. Essential oil percentage increased under FC and GA treatments compared with the control, with the highest value (2.4%) recorded under the lowest FC level (40%) combined with GA. In contrast, the highest essential oil yield (173.2 mg plant^− 1^) was obtained under 60% + GA. Statistical analysis revealed that GA had a significant effect (*P* ≤ 0.05) on essential oil percentage, whereas FC exerted a highly significant effect (*P* ≤ 0.001); however, the GA × FC interaction had no significant effect on essential oil percentage. GA had a highly significant effect on essential oil yield (*P* ≤ 0.001), whereas FC exerted a significant effect (*P* < *0.05*); in contrast, the GA × FC interaction had no significant effect on essential oil yield.

### Response of dill essential oil constituents to FC, GA, and their interaction

As presented in Tables 3 and 4, essential oil analysis identified α-phellandrene, limonene, and dill apiole as the major constituents of dill essential oil. The highest concentration of α-phellandrene (71.1%) was showed in essential oil extracted from plant exposed to FC (40%) with GA. The maximum value of dill apiole (11.9%) was recorded under the FC (40%) without or with GA treatment; while the maximum value of limonene (18.3%) was recorded with FC (80%) x GA. Although the relative abundance of individual constituents increased, decreased, or remained unchanged under FC rates and GA treatments compared with the control, no qualitative changes in essential oil composition were observed. Monoterpene hydrocarbons (MH) represented the predominant chemical class in all treatments, exceeding oxygenated monoterpenes (OM). Among the MH fraction was the dominant constituent, accounting for 80.9–88.1% of the total oil across treatments. FC and GA primarily induced quantitative rather than qualitative changes in essential oil composition, with pronounced variations in the levels of major constituents. Statistical analysis indicated that the FC × GA interaction had highly significant effect (*P* ≤ 0.001) on most dill essential oil constituents and its groups, except on α-pinene and α-phellandrene had no significant effect (Table 3). However, GA had a highly significant effect (*P* ≤ 0.001) on most dill essential oil constituents and its groups, except on α-pinene and dill ether had no significant impact (Table 4). While, FC had highly significant effects (*P* ≤ 0.001) on dill essential oil constituents and its groups (Table 4).

**Table 3:** Effect of the interaction between gallic acid x field capacity on essential oil constituents

No	Constituents (%)	RI	Gallic acid x Field capacity								F values	*P* values	MSE
			Without gallic acid				With gallic acid						
			100	80	60	40	100	80	60	40			
1	.α-Pinene	939	0.4 ± 0.1	0.5 ± 0.1	0.2 ± 0.1	0.2 ± 0.1	0.6 ± 0.1	0.5 ± 0.1	0.2 ± 0.1	0.3±0.1	2.2^ns^	0.130	0.0
2	.α-Phellandrene	1005	62.3 ± 0.3	63.1 ± 0.1	64.3 ± 0.3	65.4 ± 0.4	67.4 ± 0.4	68.9 ± 0.1	70.3 ± 0.3	71.1±0.1	2.9^ns^	0.067	0.1
3	Limonene	1031	16.6 ± 0.4^c^	17.1 ± 0.1^b^	17.3 ± 0.3^b^	16.6 ± 0.4^c^	15.4 ± 0.4^d^	18.3 ± 0.3^a^	15.1 ± 0.1^d^	14.8±0.2^e^	38.7^**^	0.000	0.1
4	.γ-Terpinene	1062	1.6 ± 0.4^b^	1.8 ± 0.2^b^	1.9 ± 0.1^b^	1.6 ± 0.4^b^	2.4 ± 0.4^a^	0.4 ± 0.1^c^	0.6 ± 0.1^c^	0.1±0.0^c^	26.5^**^	0.000	0.1
5	Dill ether	1183	2.4 ± 0.4^a^	1.1 ± 0.1^bc^	0.2 ± 0.1^e^	1.1 ± 0.1^bc^	1.6 ± 0.4^b^	0.7 ± 0.1^cd^	0.9 ± 0.1^cd^	1.4±0.4^b^	10.3^**^	0.001	0.1
6	Neomenthol	1570	1.9 ± 0.1^b^	2.2 ± 0.2^a^	2.1 ± 0.1^a^	1.4 ± 0.4^c^	1.8 ± 0.2^b^	0.2 ± 0.1^d^	0.2 ± 0.1^d^	0.1±0.0^d^	32.7^**^	0.000	0.0
**7**	Dill apiole	1685	11.2 ± 0.2^c^	11.7 ± 0.2^ab^	11.8 ± 0.2^a^	11.9 ± 0.1^a^	9.5 ± 0.5^d^	9.3 ± 0.3^d^	11.3 ± 0.3^bc^	11.9±0.1^a^	25.3^**^	0.000	0.1
8	Carvone	1242	3.3 ± 0.3^a^	2.3 ± 0.3^b^	2.1 ± 0.1^b^	0.5 ± 0.1^ef^	0.9 ± 0.1^de^	1.5 ± 0.5^c^	1.1 ± 0.1^cd^	0.2±0.1^f^	20.2^**^	0.000	0.1
Monoterpene hydrocarbons (1–4)			80.9 ± 0.1^f^	82.5 ± 0.5^e^	83.7 ± 0.3^d^	84.8 ± 0.2^c^	85.9 ± 0.1^b^	88.1 ± 0.1^a^	86.2 ± 0.2^b^	86.3±0.3^b^	85.7^**^	0.000	85.7^**^
Oxygenated monoterpenes (5–8)			18.8 ± 0.2^a^	17.3 ± 0.3^b^	16.2 ± 0.2^c^	14.9 ± 0.1^d^	13.8 ± 0.2^e^	11.7 ± 0.3^f^	13.5 ± 0.5^e^	13.6±0.4^e^	66.9^**^	0.000	66.9^**^

F-statistics: ^*^ and ^**^ indicate significance at *P* ≤ 0.05 and *P* ≤ 0.001, respectively; all values are presented as mean ± SD. MSE, mean squared error

**Table 4:** Effect of gallic acid or field capacity on essential oil constituents

No	Constituents	RI	Overall field capacity (%)				Overall gallic acid					
			100	80	60	40	Without	With	F values		*P* values	
									Gallic acid	Field capacity	Gallic acid	Field capacity
1	.α-Pinene	939	0.6 ± 0.2^a^	0.5 ± 0.1^a^	0.2 ± 0.1^b^	0.3 ± 0.1^b^	0.3 ± 0.2	0.4 ± 0.2	4.4^ns^	13.5^**^	0.053	0.000
2	.α-Phellandrene	1005	64.9 ± 2.8^d^	66.0 ± 3.2^c^	67.3 ± 3.3^b^	68.3 ± 3.1^a^	63.8 ± 1.3^b^	69.4 ± 1.5^a^	2471.4^**^	171.2^**^	0.000	0.000
3	Limonene	1031	16.0 ± 0.7^b^	17.7 ± 0.7^a^	16.2 ± 1.2^b^	15.7 ± 1.0^b^	16.9 ± 0.4^a^	15.9 ± 1.5^b^	66.7^**^	52.9^**^	0.000	0.000
4	.γ-Terpinene	1062	2.0 ± 0.6^a^	1.1 ± 0.8^b^	1.3 ± 0.7^b^	0.9 ± 0.9^c^	1.7 ± 0.3^a^	0.9 ± 0.8^b^	63.1^**^	21.4^**^	0.000	0.000
5	Dill ether	1183	2.0 ± 0.6^a^	0.9 ± 0.2^c^	0.6 ± 0.4^d^	1.3 ± 0.3^b^	1.2 ± 0.8	1.2 ± 0.5	0.2^ns^	34.8^**^	0.641	0.000
6	Neomenthol	1570	1.9 ± 0.2^a^	1.2 ± 1.1^b^	1.2 ± 1.0^b^	0.8 ± 0.8^c^	1.9 ± 0.4^a^	0.6 ± 0.4^b^	301.0^**^	35.5^**^	0.000	0.000
**7**	Dill apiole	1685	10.4 ± 1.0^c^	10.5 ± 1.3^c^	11.6 ± 0.4^b^	11.9 ± 0.1^a^	11.7 ± 0.3^a^	10.5 ± 1.2^b^	111.4^**^	49.5^**^	0.000	0.000
8	Carvone	1242	2.1 ± 1.3^a^	1.9 ± 0.6^a^	1.6 ± 0.6^b^	0.4 ± 0.2^c^	2.1 ± 1.1^a^	0.9 ± 0.5^b^	126.6^**^	61.7^**^	0.000	0.000
Monoterpene hydrocarbons (1–4)			83.4 ± 2.7^c^	85.3 ± 3.1^a^	85.0 ± 1.4^b^	85.6 ± 0.9^a^	83.0 ± 1.5^b^	86.6 ± 0.9^a^	1184.2^**^	82.8^**^	0.000	0.000
Oxygenated monoterpenes (5–8)			16.3 ± 2.7^a^	14.5 ± 3.1^bc^	14.9 ± 1.5^b^	14.3 ± 0.8^c^	16.8 ± 1.5^a^	13.2 ± 0.9^b^	888.2^**^	56.1^**^	0.000	0.000

F-statistics: ^*^ and ^**^ indicate significance at *P* ≤ 0.001; all values are presented as mean ± SD

### Changes in the phenolic content of dill in response to FC, GA, and their interaction

As illustrated in Table 5 total phenolic content was affected by FC levels, GA application, and their interaction. An overall increasing trend in phenolic accumulation was observed with reducing FC, particularly when combined with GA. The maximum total phenolic content (7.8 mg g^− 1^) was obtained under the FC (40%) + GA treatment, while the minimum value (3.6 mg g^− 1^) was recorded in plants grown under full FC without GA application. These results indicate that GA application under FC levels plays a key role in enhancing phenolic biosynthesis. Furthermore, the combined treatment proved more effective than individual applications, suggesting a synergistic effect between FC and GA in promoting phenolic accumulation. The effects of FC and GA on total phenolic content were highly significant (*P* ≤ *0.001*), while their interaction (FC × GA) was non-significant.

**Table 5:** Effect of gallic acid and / or field capacity on chemical contents

Field capacity (%)	Gallic acid (mg L^− 1^)	Phenols	Flavonoids	DPPH radical scavenging activity	Crude protein	Carbohydrates
		(mg g^− 1^)		(%)		
100	Without gallic acid	3.6 ± 0.5	1.5 ± 0.4	25.3 ± 2.0	16.3 ± 2.3	16.4 ± 1.4
80		4.2 ± 0.4	2.1 ± 0.2	27.3 ± 2.4	14.4 ± 1.9	18.5 ± 1.0
60		4.8 ± 0.5	2.4 ± 0.3	30.5 ± 2.3	13.1 ± 2.3	20.6 ± 1.5
40		6.7 ± 0.5	2.9 ± 0.4	32.7 ± 1.7	11.9 ± 1.9	21.9 ± 1.7
Overall without gallic acid		4.8 ± 1.3^b^	2.2 ± 0.6^b^	29.0 ± 3.5^b^	13.9 ± 2.4^b^	19.4 ± 2.5^b^
100	With gallic acid	4.7 ± 0.5	2.2 ± 0.3	27.5 ± 0.8	19.4 ± 1.9	19.0 ± 1.2
80		5.0 ± 0.4	2.4 ± 0.3	30.6 ± 1.9	17.5 ± 2.3	21.7 ± 1.9
60		6.1 ± 0.4	2.7 ± 0.4	33.8 ± 2.1	15.6 ± 1.9	23.9 ± 1.7
40		7.8 ± 0.5	3.1 ± 0.3	36.2 ± 1.8	13.8 ± 1.9	26.1 ± 1.1
Overall with gallic acid		5.9 ± 1.3^a^	2.6 ± 0.5^a^	32.0 ± 3.7^a^	16.6 ± 2.8^a^	22.7 ± 3.0^a^
100	Overall field capacity	4.2 ± 0.7^c^	1.9 ± 0.5^c^	26.4 ± 1.8^c^	17.9 ± 2.5^a^	17.7 ± 1.8^c^
80		4.6 ± 0.6^c^	2.3 ± 0.3^bc^	29.0 ± 2.6^b^	15.9 ± 2.5^ab^	20.1 ± 2.2^b^
60		5.5 ± 0.8^b^	2.6 ± 0.4^b^	32.2 ± 2.6^a^	14.4 ± 2.3^bc^	22.3 ± 2.3^a^
40		7.3 ± 0.7^a^	3.0 ± 0.3^a^	34.5 ± 2.5^a^	12.8 ± 2.0^c^	24.0 ± 2.6^a^
F values						
Gallic acid		36.7^**^	7.7^*^	15.7^**^	10.3^*^	31.7^**^
Field capacity		59.6^**^	12.9^**^	20.7^**^	6.7^**^	21.3^**^
Gallic acid x Field capacity		0.3^ns^	0.**7**^**ns**^	0.1^ns^	0.1^ns^	0.3^ns^
P values						
Gallic acid		0.000	0.014	0.001	0.005	0.000
Field capacity		0.000	0.000	0.000	0.000	0.000
Gallic acid x Field capacity		0.798	0.582	0.932	0.1	0.816
MSE		0.2	0.1	3.6	4.1	2.1

F-statistics: ^*^ and ^**^ indicate significance at *P* ≤ 0.05 and *P* ≤ 0.001, respectively; all values are presented as mean ± SD. MSE, mean squared error

### Effects of FC, GA, and their combined application on flavonoid content of dill

As presented in Table 5, total flavonoid content was influenced by FC, GA, and their interaction. Decreasing FC levels in combination with GA application enhanced flavonoid accumulation compared with the untreated plants. The highest total flavonoid content (3.1 mg g^− 1^) was recorded under the FC (40%) + GA treatment, whereas the lowest value (1.5 mg g^− 1^) was observed in plants grown under FC without GA application. Overall, the combined application of FC and GA was more effective in promoting flavonoid accumulation than either treatment alone. Analysis of variance showed that GA had a significant effect on total flavonoid content (*P* < *0.05*), while FC exerted a highly significant effect (*P* ≤ 0.001). However, the GA × FC interaction had no significant effect on total flavonoid content.

### Effects of FC, GA, and their combined application on the DPPH radical scavenging activity of dill

As presented in Table 5, free radical scavenging activity was significantly influenced by FC, GA, and their interaction. Reducing FC levels in combination with GA application enhanced free radical scavenging activity compared with the untreated control. The highest DPPH radical scavenging activity (36.2%) was recorded under the 40% FC + GA treatment, whereas the lowest value (25.3%) was observed in plants grown under 100% FC without GA application. Overall, the combined application of reduced FC and GA was more effective in enhancing DPPH radical scavenging activity than either treatment applied individually. Analysis of variance revealed highly significant main effects of FC and GA (*P* ≤ 0.001), whereas their interaction (FC × GA) was non-significant.

### Effects of FC, GA, and their interaction on crude protein content of dill

As presented in Table 5, crude protein content was influenced by FC, GA application, and their interaction. Overall, crude protein content declined progressively with decreasing FC levels, whereas GA application increased crude protein content under all moisture regimes. The highest crude protein content (19.4%) was recorded in plants irrigated at full FC and treated with GA, while the lowest value (11.9%) was observed at 40% FC without GA application. Analysis of variance revealed highly significant main effects of FC and GA (*P* ≤ *0.001*). Analysis of variance indicated that GA significantly affected crude protein content (*P* < *0.05*), whereas FC had a highly significant effect (*P* ≤ 0.001). In contrast, the GA × FC interaction did not significantly affect crude protein content.

### Influence of FC, GA, and their interaction on total carbohydrate content of dill

According to the data presented in Table 5, total carbohydrate content was significantly affected by FC, GA application, and their interaction. Decreasing FC levels promoted carbohydrate accumulation, and the stimulatory effect was further intensified by GA treatment. Consequently, plants grown under 40% FC combined with GA exhibited the highest total carbohydrate content (26.1%), whereas those grown under full FC without GA showed the lowest value (16.4%). Analysis of variance indicated highly significant effects of FC and GA (*P* ≤ *0.001*), with a no significant FC × GA interaction (*P* > 0.05).

### Influence of FC, GA, and their interaction on proline content of dill

As presented in Table 6, proline content was influenced by FC, GA application, and their interaction. Proline accumulation increased progressively as FC declined from full irrigation to 40%, indicating a strong response to water deficit. Exogenous application of GA further enhanced proline accumulation under each moisture regime. Accordingly, the highest proline content (10.4 µmol g^− 1^) was achieved under 40% FC with GA application, whereas the lowest value (4.7 µmol g^− 1^) was recorded in plants receiving full FC without GA. Analysis of variance demonstrated highly significant main effects of FC and GA (*P* < *0.001*), and a no significant FC × GA interaction (*P* > 0.05).

**Table 6:** Effect of gallic acid and / or field capacity on chemical contents

Field capacity (%)	Gallic acid (mg L^− 1^)	Proline	Antioxidant enzymes		
			Catalase	Superoxide dismutase	Peroxidase
		(µmol g^− 1^)	(unit g^− 1^ FW min^− 1^)		
100	Without gallic acid	4.7 ± 0.0	0.2 ± 0.0^f^	4.7 ± 0.6	3.0 ± 0.3
80		5.9 ± 0.8	0.3 ± 0.0^e^	5.6 ± 0.4	3.5 ± 0.0
60		6.2 ± 0.2	0.3 ± 0.0^d^	5.8 ± 0.0	3.8 ± 0.1
40		8.6 ± 0.5	0.4 ± 0.0^d^	6.6 ± 0.3	3.8 ± 0.1
Overall without gallic acid		6.4 ± 1.5^b^	0.3 ± 0.1^b^	5.7 ± 0.8^b^	3.5 ± 0.4^b^
100	With gallic acid	5.7 ± 0.3	0.4 ± 0.0^cd^	7.0 ± 0.5	3.2 ± 0.1
80		8.2 ± 0.8	0.4 ± 0.0^c^	8.1 ± 0.6	3.8 ± 0.1
60		8.5 ± 0.1	0.5 ± 0.0^b^	9.2 ± 0.8	4.3 ± 0.1
40		10.4 ± 1.9	0.6 ± 0.0^a^	9.9 ± 0.6	4.5 ± 0.1
Overall with gallic acid		8.2 ± 1.9^a^	0.5 ± 0.1^a^	8.5 ± 1.2^a^	3.9 ± 0.5^a^
100	Overall field capacity	5.2 ± 0.6^c^	0.3 ± 0.1^d^	5.9 ± 1.4^d^	3.1 ± 0.2^c^
80		7.1 ± 1.0^b^	0.4 ± 0.1^c^	6.9 ± 1.5^c^	3.7 ± 0.2^b^
60		7.3 ± 0.5^b^	0.4 ± 0.1^b^	7.5 ± 1.9^b^	4.0 ± 0.3^a^
40		9.5 ± 1.6^a^	0.5 ± 0.1^a^	8.2 ± 1.8^a^	4.1 ± 0.4^a^
F values					
Gallic acid		30.2^**^	292.6^**^	181.4^**^	49.5^**^
Field capacity		27.6^**^	56.6^**^	22.2^**^	63.2^**^
Gallic acid x Field capacity		0.8^ns^	5.4^*^	1.6^ns^	2.7^ns^
P values					
Gallic acid		0.000	0.000	0.000	0.000
Field capacity		0.000	0.000	0.000	0.000
Gallic acid x Field capacity		0.537	0.009	0.225	0.077
MSE		0.7	0.0	0.3	0.0

F-statistics: ^*^ and ^**^ indicate significance at *P* ≤ 0.05 and *P* ≤ 0.001, respectively; all values are presented as mean ± SD. MSE, mean squared error

### Response of antioxidant enzyme activities to FC and GA treatments in dill

As presented in Table 6, the activities of CAT, SOD and POX were affected by FC, GA application and their interaction. Antioxidant enzyme activities increased progressively as FC levels decreased, and GA application further stimulated these activities under all moisture regimes. Plants subjected to 40% FC in combination with GA exhibited the highest CAT (0.6 unit g^− 1^ FW min^− 1^), SOD (9.9 unit g^− 1^ FW min^− 1^), and POX (4.5 unit g^− 1^ FW min^− 1^) activities, whereas the lowest values (0.2, 4.7, and 3.0 unit g^− 1^ FW min^− 1^, respectively) were recorded under full FC without GA. Analysis of variance showed that GA or FC exerted a highly significant effect on antioxidant enzyme activities (*P* ≤ 0.001). In contrast, FC × GA interactions were significant at the 5% probability level (*P* < *0.05*) for CAT, whereas the effect of FC on SOD and POX activity no significant.

### Effects of FC and GA on N, P, and K accumulation in dill

As shown in Table 7, nutrient contents were influenced by FC, GA application, and their interaction. Water deficit caused a progressive reduction in the contents of N, P, and K, whereas exogenous application of GA mitigated this decline under all moisture regimes. Plants receiving full FC together with GA accumulated the greatest concentrations of N (3.1%), P (0.6%), and K (1.5%), while those subjected to 40% FC without GA exhibited the lowest corresponding values (1.9%, 0.2%, and 0.9%, respectively). Analysis of variance indicated that FC and GA had significant main effects on N and P concentrations (*P* < 0.05), whereas their interaction (FC × GA) was not significant. In contrast, K concentration was not significantly affected by FC, GA, or their interaction.

**Table 7:** Effect of gallic acid and / or field capacity on the values of minerals

Field capacity (%)	Gallic acid (mg L^− 1^)	Mineral contents			Mineral uptakes		
		(%)			mg plant^− 1^		
		Nitrogen	Phosphorus	Potassium	Nitrogen	Phosphorus	Potassium
100	Without gallic acid	2.6 ± 0.4	0.4 ± 0.1	1.3 ± 0.2	208.2 ± 36.9	32.2 ± 9.7	103.7 ± 15.4
80		2.3 ± 0.3	0.4 ± 0.1	1.2 ± 0.2	150.7 ± 19.8	26.1 ± 6.4	77.9 ± 2.7
60		2.1 ± 0.4	0.3 ± 0.1	1.1 ± 0.2	125.0 ± 16.9	17.6 ± 4.5	66.5 ± 17.6
40		1.9 ± 0.3	0.2 ± 0.1	0.9 ± 0.2	90.3 ± 6.1	9.8 ± 5.7	42.6 ± 5.7
Overall without gallic acid		2.2 ± 0.4^b^	0.3 ± 0.1^b^	1.1 ± 0.2	143.6 ± 49.0^b^	21.4 ± 10.6^b^	72.7 ± 25.2^b^
100	With gallic acid	3.1 ± 0.3	0.6 ± 0.1	1.5 ± 0.4	348.5 ± 34.0	67.5 ± 12.0	168.3 ± 45.4
80		2.8 ± 0.4	0.5 ± 0.1	1.3 ± 0.2	265.4 ± 58.4	47.6 ± 13.8	123.4 ± 30.3
60		2.5 ± 0.3	0.4 ± 0.1	1.2 ± 0.2	220.8 ± 45.3	34.9 ± 8.3	107.3 ± 33.2
40		2.2 ± 0.3	0.3 ± 0.1	1.1 ± 0.3	129.0 ± 17.7	18.0 ± 7.0	65.2 ± 18.4
Overall with gallic acid		2.7 ± 0.4^a^	0.5 ± 0.1^a^	1.3 ± 0.3	240.9 ± 90.0^a^	42.0 ± 21.0^a^	116.0 ± 47.9^a^
100	Overall field capacity	2.9 ± 0.4^a^	0.5 ± 0.1^a^	1.4 ± 0.3	278.4 ± 83.1^a^	49.8 ± 21.7^a^	136.0 ± 46.6^a^
80		2.6 ± 0.4^ab^	0.5 ± 0.1^ab^	1.3 ± 0.2	208.1 ± 73.9^b^	36.9 ± 15.2^b^	100.7 ± 31.5^b^
60		2.3 ± 0.4^bc^	0.4 ± 0.1^bc^	1.2 ± 0.2	172.9 ± 60.7^b^	26.3 ± 11.2^b^	86.9 ± 32.6^b^
40		2.1 ± 0.3^c^	0.3 ± 0.1^c^	1.0 ± 0.2	109.7 ± 24.3^c^	13.9 ± 7.2^c^	53.9 ± 17.4^c^
F values							
Gallic acid		10.3^*^	9.4^*^	2.3^ns^	50.5^**^	31.8^**^	18.0^**^
Field capacity		6.7^*^	7.4^*^	2.9^ns^	26.4^**^	17.6 ^**^	11.1^**^
Gallic acid x Field capacity		0.1^ns^	0.4^ns^	0.1^ns^	2.5^ns^	2.4^ns^	0.7^ns^
*P* values							
Gallic acid		0.005	0.007	0.149	0.000	0.000	0.001
Field capacity		0.004	0.003	0.068	0.000	0.000	0.000
Gallic acid x Field capacity		0.940	0.772	0.967	0.098	0.106	0.557
MSE		0.1	0.0	0.1	1126.1	79.8	626.1

F-statistics: * and ** indicate significance at *P* ≤ 0.05 and *P* ≤ 0.001, respectively; all values are presented as mean ± SD. MSE, mean squared error

As presented in Table 7, the uptake of N, P and K were affected by FC, GA application, and their interaction. A progressive decline in nutrient uptake was observed with decreasing FC levels especially at 40 and 60% FC, whereas GA application consistently improved N, P, and K uptake under all moisture regimes. The highest uptake of N (348.5 mg plant^− 1^), P (67.5 mg plant^− 1^), and K (168.3 mg plant^− 1^) was recorded in plants grown under 100% FC with GA application, while the lowest values (90.3, 9.8, and 42.6 mg plant^− 1^, respectively) were observed under 40% FC without GA. Analysis of variance revealed highly significant effects (*P* ≤ *0.001*) of FC or GA, and non-significant of their interactions on N, P, and K uptakes.

## Discussion

Obtained results indicated that FC-deficit reduced growth parameters; the decline in plant growth characteristics under reduced FC levels is primarily associated with the detrimental effects of drought stress on cellular and physiological processes. Moisture deficiency may reduce cell turgor and potentially restrict cell division, elongation, and expansion, particularly in stems and leaves, which could contribute to reduced plant growth, leaf development, and biomass accumulation [50]. Cell expansion is among the earliest and most drought-sensitive physiological processes, and its inhibition is commonly reflected in smaller leaves and reduced vegetative growth [51]. Furthermore, drought limits the availability of water and metabolites required for normal growth, leading to reduced photosynthetic activity and impaired synthesis of structural compounds necessary for cell development [30]. The reduction in leaf size also decreases the photosynthetically active surface available for light interception, thereby lowering carbon assimilation and further suppressing plant growth and productivity [51]. In addition to restricting growth through moisture limitation, drought stress induces severe oxidative damage by enhancing the production of reactive ROS, including superoxide radicals, hydrogen peroxide, and hydroxyl radicals. Under normal conditions, ROS are efficiently detoxified by enzymatic and non-enzymatic antioxidant defense systems. However, under prolonged or severe drought, ROS production exceeds the scavenging capacity of these defense mechanisms, resulting in oxidative stress and the activation of proteases and endonucleases [52]. The excessive accumulation of ROS causes lipid peroxidation of cellular membranes disrupts membrane permeability and stability, inhibits the activity of metabolic enzymes, impairs the photosynthetic machinery, damages nucleic acids, and ultimately accelerates cellular deterioration, all of which contribute to the inhibition of plant morphological development [53]. The adverse effects of drought on plant physiology and growth have been extensively documented. Misra [54] reported that water stress significantly reduced CO₂ exchange rate, total assimilatory area, chlorophyll and carotenoid concentrations, as well as fresh and dry biomass in Japanese mint. Likewise, Slama [55] demonstrated that drought-induced reductions in photosynthetic activity limited dry matter accumulation. Collectively, these findings suggest that the suppression of plant growth under drought conditions may be associated with physiological and metabolic alterations, potentially including impaired cell expansion, reduced photosynthetic efficiency, and oxidative stress, which could contribute to lower plant productivity.

Exposure to reduced FC resulted in marked declines in plant growth, photosynthetic pigment levels, essential oil yield, and the accumulation of N, P, K, and protein, while promoting essential oil accumulation and enhancing phenolic and flavonoid contents, DPPH radical scavenging activity, proline and carbohydrate accumulation, and antioxidant enzyme activities. The decline in photosynthetic pigments under low FC is likely associated with drought-induced impairment of chloroplast structure and suppression of chlorophyll biosynthesis. Moreover, drought stress enhances proline accumulation as part of the plant’s adaptive response. Because proline and photosynthetic pigments share a common metabolic precursor, preferential allocation of this precursor toward proline production may limit pigment biosynthesis and contribute to the observed reduction in photosynthetic pigments [55]. Moisture deficiency also increased essential oil content, possibly through stimulation of glandular trichome formation and enhancement of the biosynthetic and storage capacity for essential oils [56]. However, the concomitant reduction in plant dry biomass resulted in lower essential oil yield per plant despite the higher oil concentration, indicating that the decline in oil yield under drought conditions was primarily associated with reduced vegetative growth and dry matter accumulation [56]. Variations in essential oil content and composition among FC treatments may further reflect drought-induced alterations in the enzymatic activities and metabolic pathways involved in essential oil biosynthesis [57, 58].

The accumulation of phenolic and flavonoid compounds under low FC may represent an important non-enzymatic antioxidant response to moisture-deficit stress. These compounds contribute to ROS and free-radical scavenging, thereby reducing oxidative damage and supporting plant tolerance to drought [53, 59]. Similarly, increased carbohydrate accumulation may contribute to stress adaptation by providing readily available carbon and energy sources, maintaining metabolic homeostasis, and supporting recovery after stress alleviation [54, 59]. The marked accumulation of proline in dill leaves under reduced FC further indicates its role in osmotic adjustment and drought adaptation. Proline can function as a compatible osmolyte and as a reserve of carbon, nitrogen, and energy, thereby contributing to cellular protection and tissue recovery under drought conditions [60]. In parallel, enhanced SOD, CAT, and POX activities under low FC indicate activation of the enzymatic antioxidant defense system. SOD converts superoxide radicals (O₂•⁻) into H₂O₂, while CAT and POX subsequently contribute to H₂O₂ detoxification, thereby limiting ROS accumulation and oxidative damage to membranes, proteins, and photosynthetic components [58, 61]. Thus, the coordinated enhancement of enzymatic and non-enzymatic antioxidant defenses, together with osmotic adjustment through proline and carbohydrate accumulation, likely contributed to the ability of dill plants to maintain cellular homeostasis under moisture-deficit conditions.

In contrast, the reductions in N, P, K, and protein contents under reduced FC may be attributed to restricted nutrient uptake and transport, impaired metabolic activity, and reduced dry matter accumulation under water-deficit conditions [58, 60]. Collectively, these responses demonstrate that FC deficit imposed substantial restrictions on growth, photosynthetic performance, nutrient accumulation, and essential oil yield, while simultaneously activating biochemical mechanisms associated with drought adaptation, including enhanced secondary metabolite accumulation, osmotic adjustment, and antioxidant defense [58, 60].

Moisture deficit adversely affects plant growth and productivity by disrupting physiological and biochemical processes, including photosynthesis, nutrient uptake, and cellular metabolism. In response to drought stress, plants accumulate a wide range of protective metabolites, such as phenolic compounds, flavonoids, proline, soluble sugars, and antioxidant enzymes, which collectively contribute to stress tolerance by maintaining osmotic balance and minimizing oxidative damage. In the present study, GA application effectively alleviated the adverse effects of moisture deficit and improved plant growth, yield, and chemical composition. These beneficial effects may be attributed to the multiple physiological and biochemical functions of GA. As a naturally occurring phenolic compound with strong antioxidant properties, GA enhances the antioxidant defense system by scavenging reactive oxygen species (ROS), preserving cellular redox homeostasis, and protecting membrane lipids from oxidative damage. This protective action is closely associated with the general role of phenolic compounds in maintaining plasma membrane stability, one of the earliest cellular targets of drought-induced oxidative stress. By limiting lipid peroxidation and regulating cellular redox balance, GA helps preserve membrane integrity, sustain calcium homeostasis, maintain nitrate uptake and plasma membrane H⁺-ATPase activity, and ultimately support normal physiological functions under stress conditions [62, 63]. Furthermore, GA may be associated with improved maintenance **of** the photosynthetic apparatus and chlorophyll status, which could contribute to the maintenance of photosynthetic activity and carbon assimilation. It may also promote the accumulation of osmoprotective compounds, particularly proline, which may contribute to osmotic adjustment and the stabilization of cellular structures under water-limited conditions. In addition, the observed improvement in nutrient accumulation following GA application may be associated with enhanced nutrient acquisition and utilization, which could potentially support carbohydrate production and other metabolic processes involved in plant growth. These responses may contribute to improved overall plant growth and greater biomass accumulation. Moreover, the increased accumulation of primary and secondary metabolites observed following GA application may contribute to the plant’s ability to cope with moisture deficit. Collectively, these findings suggest that GA may alleviate some of the detrimental effects of moisture deficit through coordinated physiological and biochemical responses, thereby contributing to improved plant growth, productivity, and chemical quality under water-limited conditions. However, the specific mechanisms underlying these responses remain to be elucidated, as photosynthetic efficiency, carbon assimilation, and the molecular or enzymatic pathways involved were not directly assessed in the present study [64].

Essential oil is the principal bioactive product of dill and the main determinant of its medicinal and commercial value. In the present study, GA enhanced essential oil content and yield, which may be attributed to its ability to stimulate physiological and metabolic processes associated with plant growth and secondary metabolism. GA application may be associated with improved photosynthetic performance and assimilate availability and may potentially influence enzymatic pathways involved in terpenoid biosynthesis, which could contribute to the greater accumulation of essential oil constituents observed in the present study. However, these mechanisms were not directly assessed in the present study [58, 65]. Moreover, as a phenolic compound, GA activates the phenylpropanoid pathway, promoting the production of antioxidant metabolites that improve plant adaptation to drought stress [66]. GA may also potentially influence the development of secretory structures and the activity of terpene-synthesizing enzymes, which could contribute to terpenoid biosynthesis and accumulation in dill essential oil. However, these structural and enzymatic mechanisms were not directly assessed in the present study [67, 68]. Several studies have demonstrated that exogenous application of phenolic compounds enhances essential oil content and yield, and the accumulation of major essential oil constituents and terpene groups, such as monoterpene hydrocarbons, oxygenated monoterpenes, sesquiterpene hydrocarbons, and oxygenated sesquiterpenes, in aromatic plants. These beneficial effects may be associated with physiological and metabolic changes that could potentially influence photosynthetic performance and terpene biosynthesis, thereby contributing to greater essential oil production and changes in essential oil composition. However, these underlying mechanisms were not directly assessed in the present study [69, 70].

In the present study, the beneficial effects of GA were particularly evident under moisture-deficit conditions, suggesting that GA may enhance the ability of dill plants to cope with reduced water availability. The observed responses, including improvements in growth, antioxidant-related traits, and metabolite accumulation, may reflect enhanced physiological and biochemical adjustment under water limitation. Our findings are consistent with recent studies demonstrating that GA functions as an effective phytostimulant in aromatic and other plants under drought stress. For example, exogenous GA has been reported to improve plant growth, essential oil yield and quality, terpene accumulation, antioxidant capacity, and drought acclimation in spearmint [27, 71]. These findings provide supporting evidence for the responses observed in the present study, although the specific mechanisms involved in dill remain to be elucidated. Similar responses have also been associated with general drought-adaptation processes in medicinal and aromatic plants, including antioxidant defense, osmotic adjustment, and secondary metabolite accumulation [72]. Thus, the present findings suggest that GA may contribute to drought tolerance in dill through coordinated physiological and biochemical responses, However, these proposed mechanisms should be considered tentative, as the specific molecular pathways underlying the observed responses were not directly investigated in the present study. GA pretreatment has also been reported to improve drought tolerance in wheat by enhancing antioxidant defense and reducing oxidative damage, as indicated by increased antioxidant enzyme activities and proline and phenolic accumulation, together with reduced oxidative stress markers [73]. These findings support the present results in dill, particularly the observed enhancement of antioxidant-related responses under GA application, and suggest that GA may contribute to improved drought adaptation through coordinated physiological and biochemical responses. However, the specific mechanisms responsible for these responses in dill require further investigation. Exogenous application of GA has also been shown to alleviate abiotic stress in rice by improving plant water status, osmotic adjustment, and antioxidant defense, particularly through enhanced proline accumulation and antioxidant enzyme activities [74, 75]. These responses are consistent with the present findings in dill, where GA application improved several biochemical traits associated with stress adaptation, particularly under reduced FC. The greater responses observed under specific FC × GA combinations suggest that the effectiveness of GA may depend on the prevailing moisture conditions. However, the specific physiological and molecular mechanisms underlying these responses in dill cannot be confirmed from the present measurements and require further investigation. GA supplementation has been reported to alleviate abiotic stress in rice by improving water and ionic homeostasis and strengthening antioxidant defense, including enhanced phenolic accumulation and antioxidant enzyme activities [76, 77]. These responses are consistent with the present findings in dill, where GA application improved biochemical and antioxidant-related traits under different FC levels. In particular, the observed responses across the FC × GA treatments suggest that GA may enhance the ability of dill plants to maintain physiological and biochemical functions under moisture deficit. However, the direct contribution of specific ROS-detoxification pathways cannot be confirmed because the underlying molecular and enzymatic mechanisms were not directly investigated in the present study.

Overall, reduced FC considerably impaired plant growth, yield production, and various physiological and biochemical attributes of dill plants. However, exogenous application of GA partially alleviated these adverse effects and improved photosynthetic pigment concentration, osmolyte accumulation, and antioxidant enzyme activities in both drought-stressed and non-stressed dill plants. The magnitude of these responses varied with FC, indicating that the effectiveness of GA may depend on the prevailing moisture conditions. Therefore, exogenous GA application may represent a promising approach for improving the growth and productivity of dill under water-limited conditions, although further studies are needed to clarify the underlying mechanisms and determine its effectiveness under field conditions.

## Conclusion

Reduced FC adversely affected dill growth, photosynthetic pigments, nutrient status, and essential oil yield, while increasing proline, phenolic and flavonoid accumulation, essential oil content, and SOD, CAT, and POX activities. Foliar application of GA significantly improved the measured growth, physiological, biochemical, and antioxidant parameters under the tested water regimes. The response, however, depended on the production trait considered: maximum biomass accumulation was obtained under 100% FC + GA, whereas the highest essential oil yield was recorded with 60% FC + GA. Although water deficit increased essential oil content, the associated reduction in biomass limited total essential oil yield per plant at the more severe moisture-deficit levels. GA application partially alleviated the negative effects of water limitation and improved essential oil yield under moderate water deficit. Thus, 100% FC + GA was most favorable for biomass production, whereas 60% FC + GA was most favorable for essential oil yield, indicating that the optimal moisture regime depends on the production objective rather than representing a single optimum across all traits. However, these findings were obtained under controlled greenhouse conditions using potted plants and therefore require validation under field conditions and across different environments and irrigation regimes before broader agronomic recommendations can be made. Further studies should evaluate the economic feasibility of GA application and investigate the underlying physiological, biochemical, and molecular responses.

## Data Availability

The datasets used and/or analyzed during the current study are available from the corresponding author upon reasonable request.
